# Breast cancer incidence and survival in Scotland by socio-economic deprivation and tumour subtype

**DOI:** 10.1007/s10549-022-06632-1

**Published:** 2022-06-01

**Authors:** Ines Mesa-Eguiagaray, Sarah H. Wild, Sheila M. Bird, Linda J. Williams, David H. Brewster, Peter S. Hall, Jonine D. Figueroa

**Affiliations:** 1 Usher Institute, College of Medicine and Veterinary Medicine, University of Edinburgh, Edinburgh, UK; 2 Cambridge University’s MRC Biostatistics Unit, Cambridge, UK; 3 Cancer Research UK Edinburgh Centre, Institute of Genetics and Cancer, University of Edinburgh, Edinburgh, UK

**Keywords:** Keywords, Breast cancer, Incidence, Mortality, Subtypes, Socio-economic status

## Abstract

**Background:**

Women from socio-economically deprived areas are less likely to develop and then to survive breast cancer (BC). Whether associations between deprivation and BC incidence and survival differ by tumour molecular subtypes and mode of detection in Scotland are unknown.

**Methods:**

Data consisted of 62,378 women diagnosed with invasive BC between 2000 and 2016 in Scotland. Incidence rates and time trends were calculated for oestrogen receptor positive (ER+) and negative (ER−) tumours and stratified by the Scottish Index of Multiple Deprivation (SIMD) quintiles and screening status. SIMD is an area-based measure derived across seven domains: income, employment, education, health, access to services, crime and housing. We calculated adjusted hazard ratios (aHR [95% confidence intervals]) for BC death by immunohistochemical surrogates of molecular subtypes for the most versus the least deprived quintile. We adjusted for mode of detection and other confounders.

**Results:**

In Scotland, screen-detected ER+tumour incidence increased over time, particularly in the least deprived quintile [Average Annual Percentage Change (AAPC) = 2.9% with 95% CI from 1.2 to 4.7]. No marked differences were observed for non-screen-detected ER+tumours or ER− tumours by deprivation. BC mortality was higher in the most compared to the least deprived quintile irrespective of ER status (aHR = 1.29 [1.18, 1.41] for ER+ and 1.27 [1.09, 1.47] for ER− tumours). However, deprivation was associated with significantly higher mortality for luminal A and HER2−enriched tumours (aHR = 1.46 [1.13, 1.88] and 2.10 [1.23, 3.59] respectively) but weaker associations for luminal B and TNBC tumours that were not statistically significant.

**Conclusions:**

Deprivation is associated with differential BC incidence trends for screen-detected ER+tumours and with higher mortality for select tumour subtypes. Future efforts should evaluate factors that might be associated with reduced survival in deprived populations and monitor progress stratified by tumour subtypes and mode of detection.

## Background

Breast cancer (BC) survival has improved markedly over the last 30 years due to the introduction of mammographic screening and improvements in treatment, including targeted therapies for hormone-sensitive tumours [1]. However, socio-economic inequalities in BC survival persist in Scotland [2] and many other countries [3–6]. It is well established that BC incidence and survival differ significantly by molecular subtype [7–12]. Examining whether there are differences by deprivation for different subtypes could inform approaches to reducing inequalities through primary and secondary prevention.

Disparities by socio-economic status (SES) in BC incidence are complex and involve risk factor differences including race/ethnicity, access to healthcare and differences in the predisposition to different tumour types [13–16]. Data support risk differences by SES for different subtypes [17, 18]. The prognostic disparity by SES has been attributed to patient and clinical factors, including differences in the incidence of tumours characterized by pathologically and biologically aggressive phenotypes, the prevalence of obesity and other comorbid conditions, health-risk behaviours, access to treatment, and quality of care received [19–21].

Several studies have shown that women living in more deprived areas are more likely than those living in less deprived areas to be diagnosed with oestrogen receptor negative (ER−) and triple-negative (ER−, progesterone receptor negative (PR-), and human epidermal growth factor receptor-2 negative (HER2-)) breast cancers (TNBC) [18, 22, 23]. Race/ethnic differences in incidence of hormone negative and more aggressive BC subtypes have been observed in the US where it can be difficult to separate racial and socioeconomic disparities [24–26]. TNBC tumours are associated with early recurrence and poor survival due to lack of specific targets for commonly used adjuvant therapies [27]. It remains unclear whether differences in TNBC incidence by SES explain the observed worse prognosis of BC patients living in areas with greater socio-economic deprivation.

Greater understanding of the role of socio-economic deprivation on the incidence and survival of different subtypes of BC could inform the development of interventions aiming to reduce disparities and improve BC prognosis. Within the high-quality Scottish cancer registry, we previously showed distinct temporal trends in cancer incidence by ER status [28]. Here, we aimed to determine whether incidence (time trends) and survival by ER status and immunohistochemical (IHC) surrogate molecular BC subtypes differed by an area-based measure of SES. As a secondary aim, we investigated the effect of screening (mode of detection) on BC time trends for each SES group.

## Methods

### Study population

**Fig. 1:**
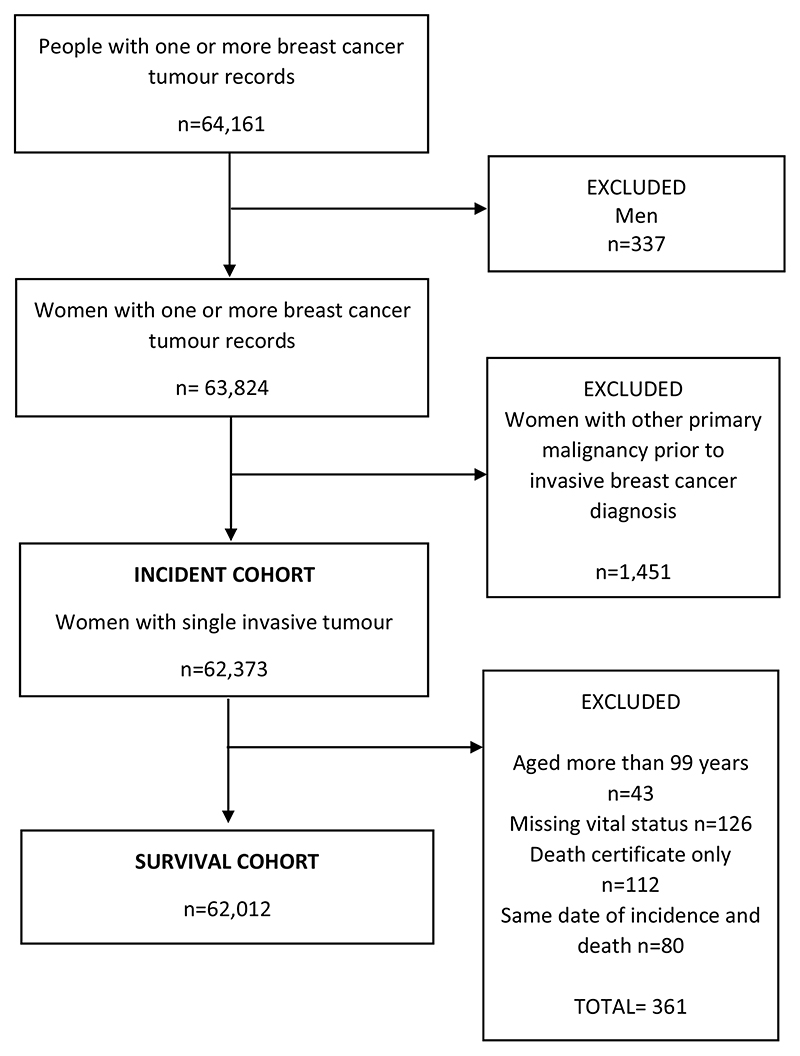
Flowchart describing incident and survival breast cancer cohorts from 2000 to 2016 in Scotland

Study data were ascertained from the Scottish Cancer Registry that covers all Scottish residents and have an overall estimate of ascertainment of BC cases greater than 98% that is independent of age [29]. The Scottish Cancer Registry was established in 1958, with electronic data linked to hospital inpatient data available from 1981. All adult women (20 years or older) diagnosed with a primary invasive BC [C50 code in the International Classification of Diseases 10^th^ Revision (ICD10)] in Scotland between 2000 and 2016 were identified. Women with other primary malignant cancers were excluded from the analysis and a single invasive BC record for each woman was selected (Fig. 1). The first invasive BC was selected as the incident cancer except when a woman had multiple primary BCs diagnosed within 6 months. In that case, the more advanced invasive cancer was selected using criteria based on grade and nodal status. The incident cohort (n = 62,373 women) was further restricted for the survival analyses: women aged more than 99 years, with missing vital status or diagnosed with BC only from death certificates were excluded from the analysis (Fig. 1). Women who had the same date of incidence and death were also excluded. The total number of excluded cases was 361 (0.6% of the total) and the final population for the survival analysis consisted of 62,012 women whose BC was diagnosed between 2000 and 2016 (Fig. 1).

### Molecular subtypes definition

ER status has been recorded in the Scottish cancer registry since 1997 for all invasive tumours diagnosed histologically, through biopsy, surgical excision or histology of nodes or metastases. The method used to assign ER status (positive or negative) to a tumour was the Allred score system and is assigned following immunohistochemical (IHC) staining for the proportion of cells that stain positively and the intensity of staining [30]. Progesterone receptor status (PR) and HER2 status were available from 2009 and were also measured using IHC. The fluorescence in situ hybridisation (FISH) test was carried out to confirm the result for HER2 status if IHC result was borderline. ER status had an 8% missing rate. However, the percentage of missing ER status decreased over time from 20% in 1997 to 2% in 2016. For that reason, the three first years (1997 to 1999) of the cohort were removed to give time to ER collection to achieve a good ascertainment rate. ER, PR and HER2 molecular markers were used as a proxy for the classification of molecular BC by mRNA expression profiling known as intrinsic molecular subtypes of BC [31]. The IHC-defined molecular subtypes were classified according to the St Gallen 2011 consensus [32]: ER+ and/or PR + and HER2− tumours were defined as luminal A, ER+ and/or PR + and HER2 + as luminal B, ER− and PR- and HER2 + as HER2− enriched and ER− and PR- and HER2− as TNBC. The Ki-67 a marker for tumour proliferation is not currently recorded in the Scottish cancer registry, which is why grade was used to further differentiate luminal A and luminal B tumours with luminal A tumours of high grade (poorly differentiated) reclassified as luminal B tumours. Missingness of IHC-defined molecular subtype was 11% for the study period.

### Deprivation definition

The Scottish Index of Multiple Deprivation (SIMD) was used as an area-based measure of SES. SIMD is based on seven domains: income, employment, health, education, crime, access to services and housing that are used to rank the 6,976 data zones in Scotland from the most deprived to the least deprived area. SIMD is often expressed in quintiles and we compared women in the most deprived fifth of areas (quintile 1) with women in the least deprived fifth of areas (quintile 5) of Scotland. SIMD is developed by the Scottish Government and linked to the Cancer Registry and other health records in Scotland. SIMD quintiles were obtained from the Scottish cancer registry and available for all women with a Scottish postcode which was 100% complete for the study data. Several SIMD versions (SIMD 2004, 2006, 2009, 2012 and 2016) were available for our study period from 2000 to 2016. The most appropriate SIMD version for each year of diagnosis was selected as recommended in the deprivation guidance for analysts [33] and a unique quintile was used for each woman.

### Screening: mode of detection

In Scotland, a national mammographic screening programme was established in 1988 and women aged 50 to 70 years old are invited to have a routine screen every three years. Women over 70 years of age are able to make appointments for continued screening. Mode of first detection was recorded in the Scottish Cancer Registry as screen-detected, not screened-detected and unknown, using electronic health records that include the screening datasets.

### Survival outcome

Breast cancer specific survival (BCSS) was the primary outcome of the survival analysis. BC deaths were derived using only the underlying (primary) cause of death as derived from death records linked to the cancer registry [34]. Date of diagnosis in the Scottish Registry is normally recorded as the date of first consultation or admission at the hospital for that cancer. This date is a definite point in time that can be verified from the records and is the most consistent and reliable date to use [35]. Duration of follow-up was defined as time from date of diagnosis of BC to the first of: date of death, 31^st^ December 2017 for women still alive at the end of the study period or embarkation date if women moved outside Scotland (within the UK). The 31st of December 2017 was selected as the end of follow-up as the data were obtained in April of 2018. Complete incidence data for the year 2016 would be expected by the end of 2017 in accordance with the United Kingdom and Ireland Association of Cancer Registries (UKIACR) guidelines. The approach taken for this analysis is similar to that described by Skyrud et al. [36] in that only ICD9 174 and ICD10 C50 codes from primary cause of death were used to derive BC-specific death. Other primary causes of death were regarded as censored observations for the calculation of BCSS.

### Statistical analysis

#### Incidence

Age-standardised incidence of BC was computed for all women living in the most and least deprived quintiles of Scotland by ER status. Counts of BC by ER status and SIMD based on a single incident BC per woman for each age and year of diagnosis were used as the numerator. The population estimates used as the denominator were mid-year population estimates for each age group (in 5-years age groups), year of diagnosis and SIMD quintile obtained from the National Records of Scotland [37]. These estimates are derived from decennial census data with adjustment for population changes in intervening years and for under-enumeration (estimated coverage was 94% in the 2011 Census) [38]. Incidence was standardised using the direct method to the European standard population (2013) in 5-year age groups. Further, incidence rates by ER status and SIMD were calculated for women of approximate screening age (50 to 69 years) and stratified by mode of detection (screen vs non-screen-detected tumours). Graphs of incidence trends were smoothed using a three-year moving average, with incidence year in the graphs representing the middle year for each three-year period (for example, year 2001 in the graph represents the average of years 2000 to 2002). The average annual percentage change (AAPC) for each ER status and the two extreme quintiles of deprivation (most and least deprived areas) was computed overall and stratified by mode of detection and is presented in the graphs with 95% Confidence Intervals (CI) [39].

### Survival analyses

Non-parametric Kaplan–Meier estimates [40] were used to estimate BCSS by ER status and the IHC-defined molecular subtypes for women by deprivation quintile. Comparisons between those in the most and least deprived areas are reported here. Five-year survival was chosen as primary endpoint as it is often used for population cancer statistics and recommended as a quality performance indicator by NHS Scotland [41]. Cox proportional hazards models [42] were fitted to investigate the association between living in the most and least deprived areas of deprivation (main exposures) and BC death amongst Scottish women with BC. Models were fitted on complete cases and stratified by ER status or IHC-defined molecular subtype to adjust for non-proportional hazards between subtypes. Models were adjusted for the following covariates: year of diagnosis, age at diagnosis, NHS Scottish region, tumour characteristics (grade, TNM stage and mode of detection), treatment regimens (surgery, radiotherapy, chemotherapy and hormone therapy) and comorbidities measured using the Charlson index of comorbidity, a measure based on hospital admission data derived from the Scottish Morbidity Records dataset [43].

## Results

### Incidence

Amongst the 62,373 BC cases diagnosed between 2000 and 2016, 18% were in the most deprived quintile and 21% were in the least deprived quintile, Table 1. The proportion of ER− cases declined over time but was slightly higher amongst women from the most deprived quintile with the highest proportion observed in 2000–2003 (21% vs 17% in the least deprived quintile). Women diagnosed with ER+tumours in the least deprived areas had slightly higher frequency of lower stage tumours (40% vs 34% stage I) but proportions of high-grade tumours (27% vs 28% for grade III) were similar to those in women from the most deprived quintile. Differences in tumour characteristics were less marked for ER−tumours, although women from the most deprived quintile had slightly lower frequencies of stage I and slightly higher frequencies of stages II and III than women in the least deprived quintile. The proportion of screen-detected tumours was higher in the least deprived quintile than in the most deprived quintile for both ER+(34% vs 28%) and ER− tumours (19% vs 15%). There were clear treatment differences between the subtypes and lower proportions of women had surgery, radiotherapy and chemotherapy in the most deprived areas of Scotland compared to the least deprived areas regardless of ER status. In contrast, proportions of women who received hormone therapy were very similar across deprivation quintiles. The descriptive characteristics for all SIMD groups stratified by ER status are presented in supplementary Table 1.

**Fig. 2:**
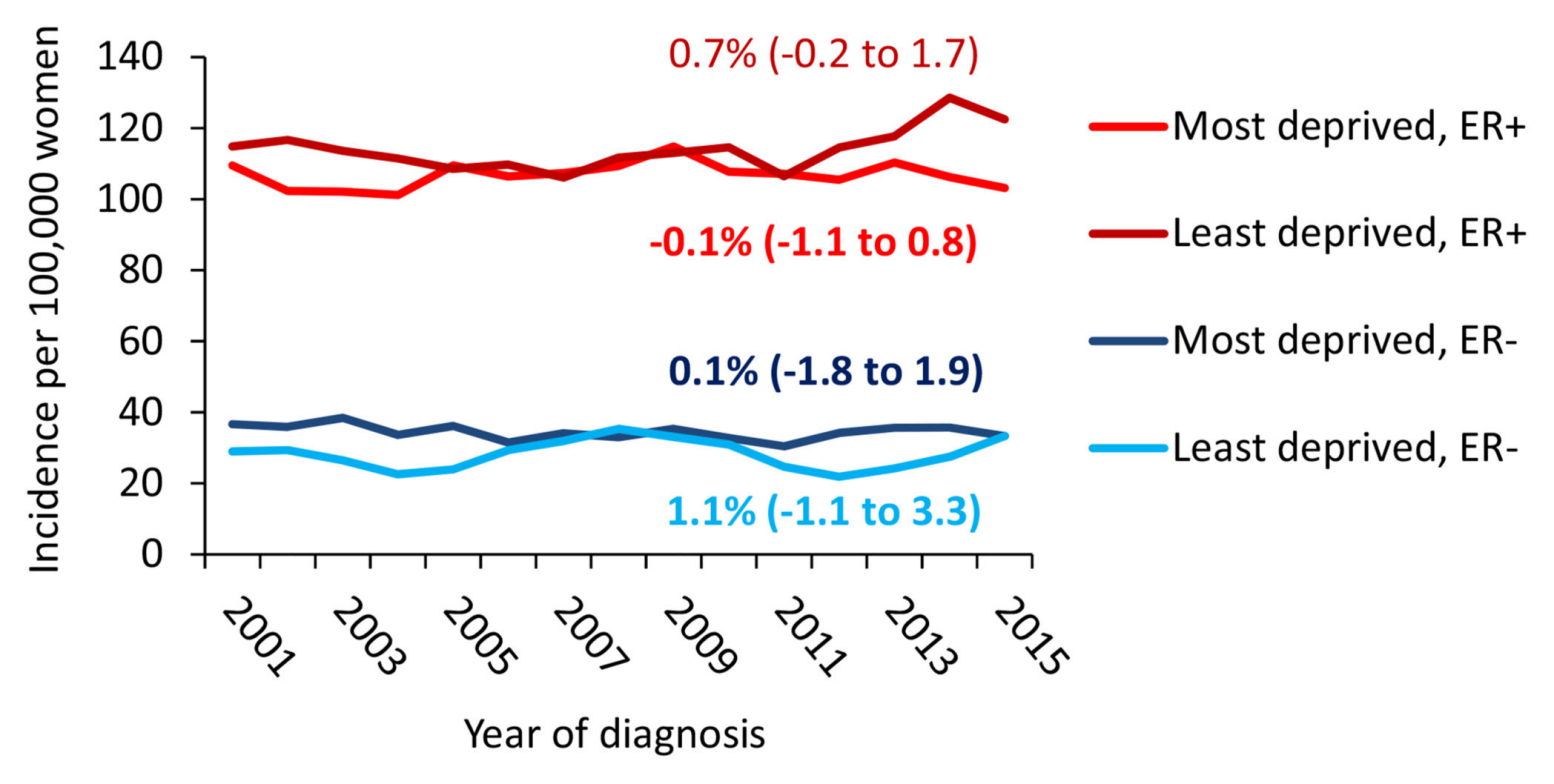
Breast cancer age-standardised incidence rates by ER status and calendar year in women living in the most and least deprived areas of Scotland for 2000–2016. Estimates in graph are AAPC (95% CI) from 2000 to 2016

Figure 2 presents temporal trends in the incidence rates from 2000 to 2016 by deprivation status. ER+tumours incidence was higher than ER− tumours incidence for all deprivation quintiles. Incidence of ER+ tumours was similar for least and most deprived quintiles with no clear increasing trend (AAPC = 0.7% (95% CI: -0.2 to 1.7) for least deprived and -0.1% (95% CI: -1.1 to 0.8) for most deprived). From 2009, ER+incidence appears to slightly increase more markedly for the least deprived quintile. For ER− tumours, incidence has remained approximately constant over time with around 40 cases per 100,000 women in the most deprived quintile; and around 30 per 100,000 women in the least deprived quintile.

**Fig. 3:**
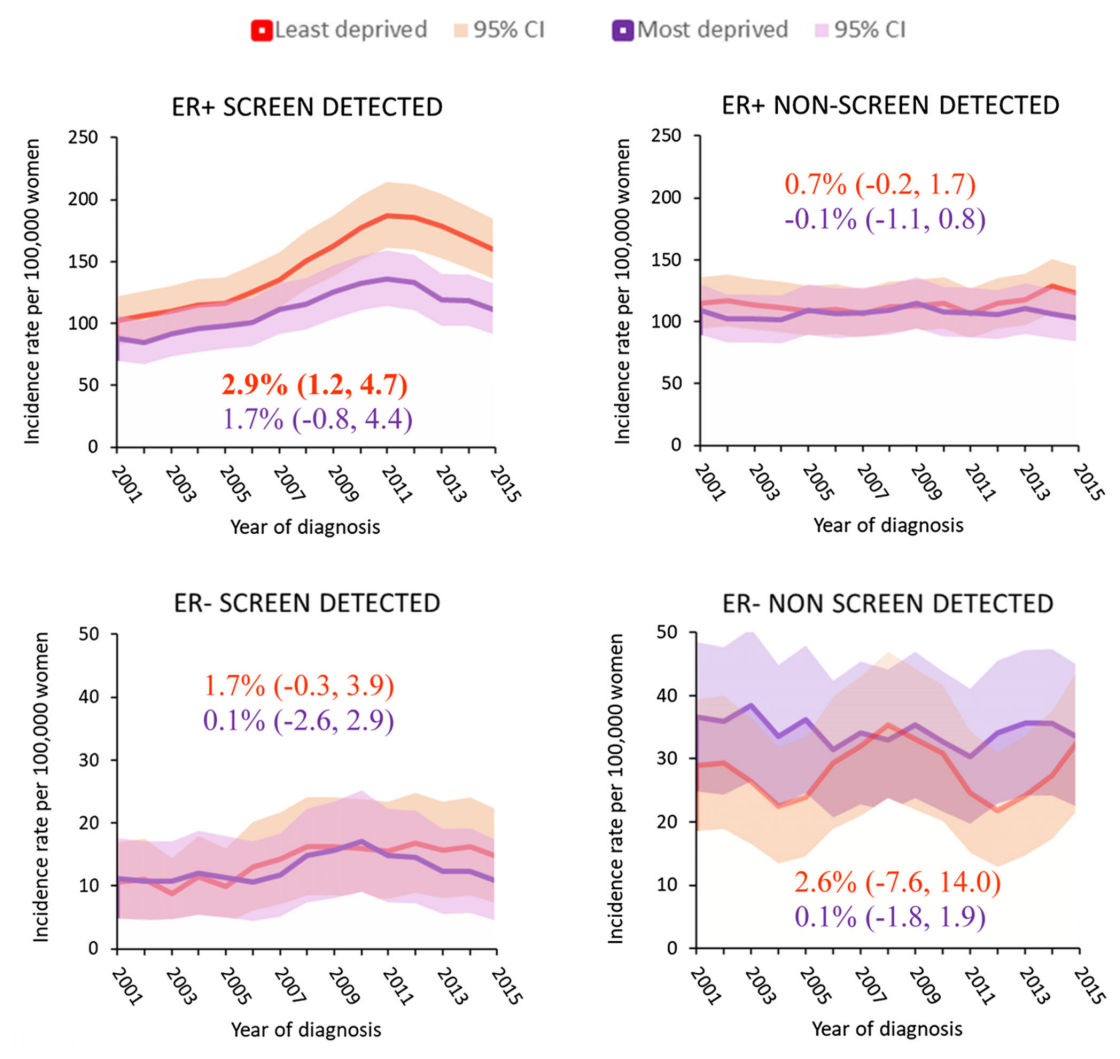
Breast cancer incidence rates in women living in most and least deprived areas of Scotland by ER status and mode of detection by calendar year for 2000–2016. Estimates in graph are AAPC (95% CI) from 2000 to 2016

Figure 3 shows that increasing incidence rates were observed for ER+screen-detected tumours in women of screening age (50 to 69 years) regardless of deprivation, although the magnitude was higher for least deprived women. The incidence pattern for this subgroup was similar to that for the whole of Scotland, with steady increases (AAPC = 2.9% [1.2, 4.7]) until early 2010s when they levelled off. In contrast, we observe no marked differences in the incidence or time trends of non-screen-detected ER+tumours by deprivation. Incidence of ER− tumours was slightly higher for non-screen-detected tumours than for screen-detected tumours with no clear differences in incidence or time trends observed by deprivation.

### Survival

Of the 62,012 women included in the survival analysis, 50,420 (81%) were followed up for 5 years or longer. In Scotland, higher proportions of women diagnosed with BC between 2000 and 2016 were alive at the end of the follow-up (2017) in the least deprived areas than in the most deprived areas, regardless of tumour subtype (Table 1, Supplementary Fig. 1). However, proportions of BC-specific deaths were similar across deprivation quintiles. Amongst women diagnosed with an ER+tumour who died during the study period, 66% died from BC in the least deprived areas compared to 64% in the most deprived areas. Proportions of deaths attributed to BC were higher for women with ER− tumours but did not differ by deprivation quintile, accounting for 82% of all deaths in the least deprived and 81% in the most deprived (Table 1).

BCSS at 5 years was highest amongst women living in the least deprived fifth of areas of Scotland diagnosed with luminal A or ER+ tumours (90.6 and 87.4% respectively) (Table 2). In contrast, women with more aggressive subtypes (ER−, HER2 enriched and TNBC) living in the most deprived fifth of areas had the lowest BCSS at 5 years with 65.1, 64.5 and 69.7% respectively. Women living in the most deprived areas had lower survival than women living in the least deprived areas for all subtypes, this difference was particularly high for women diagnosed with an ER− tumour that overexpressed HER2 (Table 2).

**Fig. 4:**
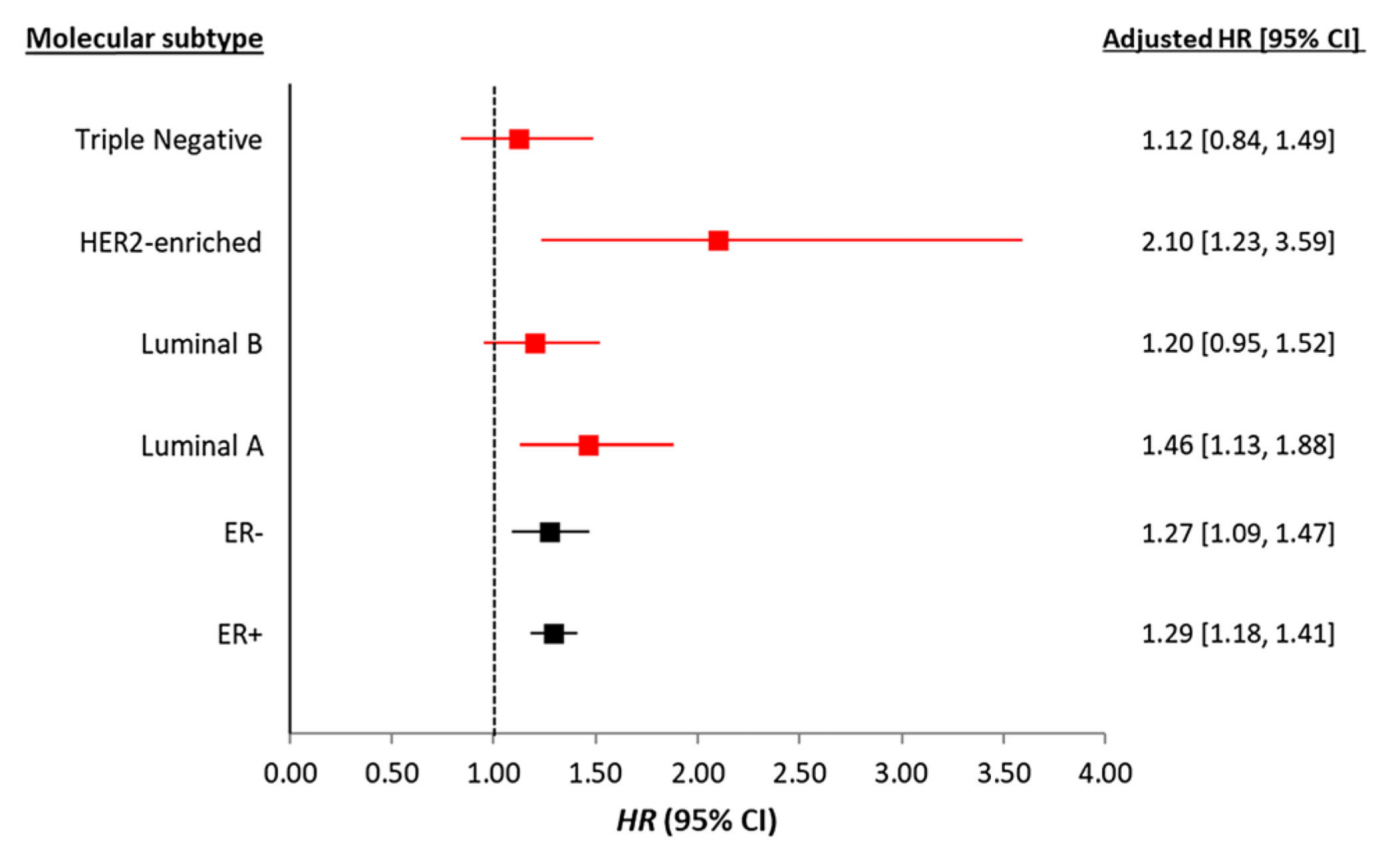
Adjusted hazard ratio (with 95% CI) for risk of BC death for women in the most compared to the least deprived quintiles by ER status (in black) and IHC-defined molecular subtypes (in red). Adjusted model has age, incidence year, NHS region, tumour characteristics (TNM stage and method of detection, treatments (surgery, radiotherapy, chemotherapy and hormone therapy) and Charlson comorbidity index. Models carried out on complete cases separately by subtype with n = 37,667 (no deaths = 5194) for ER+, n = 7598 (no deaths = 2073) for ER−, n = 12,762 (no deaths = 604) for luminal A, n = 6984 (no deaths = 808) for luminal B, n = 1029 (no deaths = 159) for HER2 enriched and n = 2,512 (no deaths = 516) for TNBC

Breast cancer specific mortality for the most compared to the least deprived quintile was similar by ER status, HR of 1.29 (95% CI: 1.18 to 1.41) and 1.27 (95% CI: 1.09 to 1.47) for ER+ and ER− tumours, respectively (Fig. 4) after adjusting for individual and tumour characteristics, treatments and comorbidities. However, deprivation showed differential associations with BC-specific mortality when using St Gallen’s IHC-defined subtypes. The highest relative risk of BC death was observed for women with the least common subtype, HER2−enriched, for whom HR was 2.1 (95% CI: 1.23 to 3.59) for those living in the most deprived areas compared to women living in the least deprived areas of Scotland. Women with luminal A tumours in the most deprived areas were 46% more likely to die of BC compared to women in the least deprived areas (Fig. 4). For women with luminal B and TNBC, there was no evidence that deprivation was associated with BC death after adjustment for other tumour characteristics, treatments and comorbidities. Fully adjusted HRs for all SIMD quintiles stratified by ER status and IHC-defined molecular subtypes are presented in Supplementary Table 2.

## Discussion

We previously reported increasing incidence of ER+ tumours by an average of 0.4%/year and decreasing incidence of ER− tumours by 2.5%/year (95% CI: − 3.9 to − 1.1%) across Scotland between 1997 and 2016 and identified that screening was a major contributor to rising incidence of ER+ tumours [28]. Here, we observed that although trends over time were similar to those previously reported regardless of deprivation, incidence increased mainly amongst women living in least deprived areas of Scotland with screen-detected ER+ tumours (AAPC of 2.9% compared to 1.6% previously reported overall) [28]. Absolute incidence for ER+ screen-detected tumours was also higher amongst the least deprived compared to the most deprived (with approximately 50 more cases per 100,000 women at the peak in 2011). Screening uptake might partially account for the differences in BC incidence observed between most and least deprived areas. This is supported by data showing uptake of BC screening in the most deprived areas of Scotland was 59.5% in 2016–2019 and 79.7% in the least deprived areas [44].

We found lower point estimates and no statistically significant association between deprivation and BC survival amongst women with the rarer subtypes of TNBC or luminal B tumours. Previous studies from Scotland and other countries have found an association between SES (at both individual and neighbourhood level) and BC mortality, with women with low SES having a higher BC mortality [22, 45, 46]. Some data show women with low SES are more likely to be diagnosed with more aggressive BC subtypes, particularly ER− and TNBC subtypes [18, 22, 23]. However, evidence of whether survival rates for subtypes differ by SES has not been investigated previously. In our multivariable analysis, deprivation was associated with statistically significantly higher BC mortality for luminal A and HER2−enriched tumour subtypes but not TNBC and luminal B tumours, for which the association was attenuated and no longer statistically significant after adjusting for screening, treatment and the Charlson index for comorbidities. Risk of BC death for HER2−enriched tumours appeared particularly high, albeit with limited power and wide confidence intervals for the most deprived areas compared to the least deprived areas, and this finding will require confirmation in other datasets to determine if it replicates. Cumulatively, our findings support associations with socio-economic deprivation in survival differ by subtypes.

Possible additional factors that could be contributing to survival differences by deprivation are alcohol intake, obesity and smoking. In Scotland, alcohol-related hospitalisation and mortality was up to 8 times higher across people from the most deprived areas. However, men and women in the most deprived areas of Scotland are less likely to drink hazardous or harmful alcohol levels than those in the least deprived areas (10% drinking at hazardous/harmful level vs 20%) [47]. Further, heavy drinking has been also consistently linked to weight gain [48]. In Scotland, obesity prevalence in women is around 30% and 20% in the most and least deprived areas, respectively [49]. Physical activity is also a noted risk factor that is approximately 20% lower in the most deprived compared to the least deprived communities in Scotland [50]. Smoking could also be a contributing factor given that prevalence was 30% compared to 9% in women in the most and least deprived areas of Scotland in 2018 [47]. The more marked differences by deprivation amongst women diagnosed with luminal A or HER2−enriched tumours than for luminal B and TNBC tumours may also be related to differences in prognosis and/or treatment adherence [51].

This study has several strengths as to our knowledge is the first study in the UK to investigate BC incidence and survival by SIMD and molecular subtypes utilising high-quality data from the Scottish cancer registry with linkage to mortality and comorbidity records. As for any observational study, the validity of our findings must be assessed in terms of potential confounding and bias. Although our analysis controlled for some potential confounders, there was no information about other risk factors, such as obesity alcohol consumption, smoking and physical activity. Another limitation is that survival rates can be affected by lead time and length biases [52].The lead time bias refers to the additional number of years added to the survival time of all women whose tumours were detected by screening [53]. Our data support that this is likely differential between deprived groups and needs to be considered in analyses of inequities with BC. On average, lead time bias is estimated to be 3 years, hence reporting 5-year survival estimates might help reduce its impact on survival rates. Length bias relates to the tumour’s presymptomatic period when it is mammographically detectable, called the sojourn time. Screening preferentially detects tumours with longer sojourn times; therefore, tumours detected through screening are slower growing and less lethal. Although we present stratified analyses by ER status and adjustment for whether tumours were detected through screening in our analysis, the potential for residual confounding remains as women who accept invitations to screening are likely to differ from women who do not attend screening in ways that may influence survival. Another limitation is the validity of BCSS analysis depends on the accuracy of cause of death as recorded in the registry which assumes that the underlying cause of death has been accurately determined for each woman. Skyrud et al. [36] compared cause-specific and relative survival estimates and found cause-specific estimates to be as reliable as relative survival estimates, particularly for common cancers. Another possible limitation is competing risks of death with women in most deprived areas being more likely to die from other causes than BC. In order to minimise competing risks of death, we restricted survival estimates to 5 years. Finally, the SIMD is an area-based measure of deprivation so it can misclassify individuals’ SES [54]. Potential misclassification is a particular risk for rural areas where the index domains particularly the ‘access’ domain fails to capture important singularities of the rural areas, such as, frequency and cost of public transport [55].

This analysis using high-quality population-based data in Scotland shows differences in incidence and prognosis between an area-based measure of SES for different molecular subtypes of BC. Determining factors that are associated with differences in the incidence and survival for different subtypes could help identify interventions for modifiable risk factors and/or identify high risk individuals to try and detect cancers earlier through screening. Tackling inequalities in BC require more detailed analyses such as ours that report incidence and survival for disease subtype characteristics [56] including stage, ER and IHC-defined molecular subtypes. These analyses should help identify where inequalities exist (or don’t) allowing cancer control programmes to focus on inequalities where they are greatest. Few studies have been able to stratify these results by subtype as well as mode of detection due to the lack of availability of such data. More detailed data on risk factors by SES, screening participation, lifestyle behaviours (e.g. smoking, physical activity, and alcohol intake), comorbid conditions, treatment and tumour subtype are required. As recently proposed [56], future analyses using modern methods of causal mediation analysis would be important to accurately estimate the contribution of potential explanatory factors for inequalities; this would provide evidence that could translate into improvements in primary and secondary prevention of BC that would have the most impact with regard to mortality.

## Supplementary Material

Supplementary File

## Figures and Tables

**Table 1:** Descriptive characteristics of women in Scotland diagnosed with invasive BC from 2000 to 2016 by extreme quintiles of the Scottish Index of Multiple Deprivation and known ER status

	Most deprived quintile *n* = 10,946 (18%)				Least deprived quintile *n* = 12,909 (21%)			
	ER+		ER−		ER+		ER−	
	*n*	%	*n*	%	*n*	%	*n*	%
	8356	[81]	1908	[19]	10,394	[85]	1861	[15]
*Age*								
<50 years	1523	[75]	517	[25]	2119	[81]	488	[19]
50–69 years	4219	[82]	920	[18]	5468	[86]	915	[14]
70 years or older	2614	[85]	471	[15]	2807	[86]	458	[14]
*Scottish region*								
North	947	[81]	216	[19]	2658	[82]	602	[18]
South East	1423	[83]	288	[17]	3679	[88]	519	[12]
West	5986	[81]	1404	[19]	4057	[85]	740	[15]
*Year of diagnosis*								
2000–2003	1649	[79]	431	[21]	2121	[83]	423	[17]
2004–2007	1856	[81]	433	[19]	2235	[86]	368	[14]
2008–2011	2100	[82]	455	[18]	2590	[85]	470	[15]
2012–2016	2751	[82]	589	[18]	3448	[85]	600	[15]
*Charlson Score Index*								
0	7810	(93)	1769	(93)	10,045	(97)	1814	(97)
1 or more	546	(7)	139	(7)	349	(3)	47	(3)
*Tumour grade*								
I	1103	(13)	25	(1)	1552	(15)	22	(1)
II	3576	(43)	244	(13)	5215	(50)	291	(16)
III	2331	(28)	1362	(71)	2797	(27)	1361	(72)
Unknown	1346	(16)	277	(15)	830	(8)	187	(10)
*Tumour stage*								
1	2850	(34)	439	(23)	4097	(40)	504	(27)
2	2966	(36)	789	(41)	3679	(35)	790	(42)
3	1151	(14)	335	(18)	1332	(13)	301	(16)
4	442	(5)	126	(7)	442	(4)	90	(5)
Unknown	947	(11)	219	(11)	844	(8)	176	(9)
*Screen detected*								
Yes	2346	(28)	279	(15)	3506	(34)	351	(19)
No	5831	(70)	1576	(83)	6781	(65)	1489	(80)
Unknown	179	(2)	53	(3)	107	(1)	21	(1)
PR status*								
Positive	3000	(69)	33	(4)	3269	(60)	53	(6)
Negative	569	(13)	780	(83)	643	(12)	717	(76)
Unknown	767	(18)	123	(13)	1543	(28)	169	(18)
HER2 status*								
Positive	487	(11)	250	(27)	599	(11)	292	(32)
Negative	3439	(79)	601	(64)	4405	(81)	572	(61)
Unknown	410	(10)	85	(9)	451	(8)	75	(8)
*Surgery*								
Yes	6846	(82)	1701	(89)	9237	(89)	1712	(92)
No	1462	(18)	196	(11)	1126	(11)	<200	(8)
Unknown	48	(0)	11	(0)	31	(0)	<10	(0)
*Radiotherapy*								
Yes	4736	(57)	1114	(58)	6780	(65)	1233	(66)
No	3163	(38)	6725	(36)	3292	(32)	564	(30)
Unknown	457	(5)	122	(6)	322	(3)	64	(4)
*Chemotherapy*								
Yes	2619	(31)	1286	(67)	3580	(34)	1330	(72)
No	5480	(66)	580	(31)	6660	(64)	510	(27)
Unknown	257	(3)	42	(2)	154	(2)	21	(1)
*Hormone therapy*								
Yes	7143	(86)	130	(7)	9050	(87)	134	(7)
No	600	(7)	1681	(88)	810	(8)	1663	(89)
Unknown	613	(7)	97	(5)	534	(5)	64	(4)
*Vital status*								
Alive	5421	(65)	1091	(57)	7888	(76)	1267	(68)
Dead	2935	(35)	817	(43)	2506	(24)	594	(32)
*BC death^*								
Yes	1872	(64)	661	(81)	1646	(66)	487	(82)
No	1063	(36)	156	(19)	860	(34)	107	(18)
*Follow-up time in yearsϯ*								
Mean (SD)	6.6	(4.6)	5.9	(4.9)	7.4	(4.7)	6.7	(4.9)

Brackets [] indicate row percentages and parenthesis () are column percentages unless indicated otherwise. N do not equal total due to missing ER status. ER status was missing in 6% of tumours diagnosed in most deprived areas and 5% of tumours in least deprived areas

* PR and HER2 figures restricted to years 2009 to 2016

^ BC death amongst those who died during follow-up. Yes = breast cancer specific death. No = other cause of death

ϯ Follow-up time amongst those who died from BC

**Table 2:** Five-year breast cancer specific survival estimates (in %) with 95% confidence intervals by molecular subtype for women living in the most and least deprived areas of Scotland whose breast cancer was diagnosed 2000–2013

Breast cancER−specific survival	Most deprived	Least deprived	Difference in proportions surviving (least minus most)
*ER+*		
Deaths/cases	1664/6159	1513/7682	
5-year BCSS (95% CI)	80.5 (79.5, 81.5)	87.4 (86.7, 88.2)	6.9 (5.7, 8.1)
*ER−*		
Deaths/cases	566/1432	420/1374	
5-year BCSS (95% CI)	65.1 (62.6, 67.6)	74.7 (72.3, 76.9)	9.6 (6.2, 13.0)
*Luminal A*		
Deaths/cases	194/1134	177/1551	
5-year BCSS (95% CI)	85.0 (82.8, 87.0)	90.6 (89.1, 92.0)	5.6 (3.1, 8.1)
*Luminal B*		
Deaths/cases	148/656	149/840	
5-year BCSS (95% CI)	81.1 (77.8, 83.9)	85.4 (82.8, 87.7)	4.3 (0.5, 8.1)
HER2−enriched		
Deaths/cases	40/112	17/113	
5-year BCSS (95% CI)	64.5 (54.8, 72.7)	85.7 (77.8, 91.0)	21.2 (10.2, 32.2)
*TNBC*		
Deaths/cases	79/252	65/243	
5-year BCSS (95% CI)	69.7 (63.5, 75.1)	74.8 (68.9, 79.8)	5.1 (-2.8, 13.0)
